# Colonic Perforation Secondary to Gallstone Impaction in the Sigmoid Colon

**DOI:** 10.1155/2023/9986665

**Published:** 2023-07-20

**Authors:** Paschalis Gavriilidis, Abhilash Paily

**Affiliations:** Department of Surgery, Colchester General Hospital, Turner Road, Colchester CO4 5JL, UK

## Abstract

**Introduction:**

Gallstone sigmoid ileus is a very rare manifestation of large bowel obstruction. Mainly, three conditions predispose the manifestation of the entity; in particular, an episode of cholecystitis causing cholecysto-colonic fistula; a large gallstone; and narrowing of the sigmoid colon secondary to diverticular disease or malignancy. *Case Report*. An 82-year-old man presented to the emergency department with a one-week history of severe constipation, tachypnoea, tachycardia, hypotension, and high lactate. Physical examination demonstrated cyanosed upper and lower extremities and palpation of the abdomen revealed signs of peritonism, abdominal distention, and guarding. Computerized tomography scan demonstrated perforation of the hollow viscus organ secondary to impaction of the large gallstone in the sigmoid colon. Laparotomy revealed sigmoid perforation and widespread feculent peritonitis. The patient underwent Hartmann's procedure. After the intervention gave concerns regarding the patient's haemodynamic stability, he was transferred to the intensive care unit. The patient passed away on the third postoperative day due to complications secondary to haemodynamic instability.

**Conclusions:**

Patients with early diagnosed uncomplicated sigmoid gallstone ileus can be managed with endoscopic mechanical lithotripsy. In case of failure, open or laparoscopic enterolithotomy can be applied. However, when patients present with complications, surgery should not be delayed. In our case, Hartmann's procedure was an absolute indication due to sigmoid perforation and widespread feculent peritonitis.

## 1. Introduction

Gallstone ileus is a rare entity, which develops in only 0.3–0.5% of patients with cholelithiasis [1]. The reported incidence rate over the period of 45 years was 30–35 cases for every one million admissions [2]. The prevalence is higher among elderly patients and 72–90% of them are women [2–4]. Gallstone ileus develops secondary to cholecysto-enteric fistula. In particular, the frequency of cholecysto-duodenal accounts for about 70% and cholecysto-colonic 10% of cases [5]. It has been reported that the commonest site of gallstone impaction is the part of the distal ileum close to the ileocaecal valve, which accounts for 60–85% of cases [6]. On the other hand, gallstone sigmoid ileus consists of only 4% of all gallstone ileus patients. This incidence rate can be interpreted that 12–15 patients per 100,000 of all patients with gallstone ileus may diagnose with sigmoid gallstone ileus [5–7]. Underlying diverticular disease complicated with stenosis is a significant causative factor of sigmoid gallstone impaction [8–11].

## 2. Case Report

An 82-year man presented to the emergency department of the general hospital with a one-week history of constipation and lower abdominal pain. On presentation, his vital signs were respiratory rate: 20 bpm, heart rate: 137 bpm, blood pressure: 89/68 mmHg, temperature:36.5°C, and O_2_ saturation:99%. Physical examination revealed cyanosed upper and lower extremities, the abdomen was distended with signs of peritonism. Laboratory results were white blood cells: 4.2, neutrophils: 0.01, C-reactive protein: 42, lactate: 7.9, urea: 9.6 mmol/L (2.5–7.8 mmol/L), creatinine: 160 mmol/L (59–104 mmol/L), total bilirubin: 27(0–21), and prothrombin time: 19. The rest of the laboratory results were within normal limits.

Past medical history, included atrial fibrillation on abixan, on February 2021, he had an episode of acute cholecystitis, and the computerized tomography (CT) scan demonstrated a large gallstone. The patient managed conservatively and on the seventh postoperative day, the patient was discharged home and scheduled for elective cholecystectomy. The patient declined the operation for personal reasons because all this period did not have any recurrence of the symptoms.

**Figure 1:**
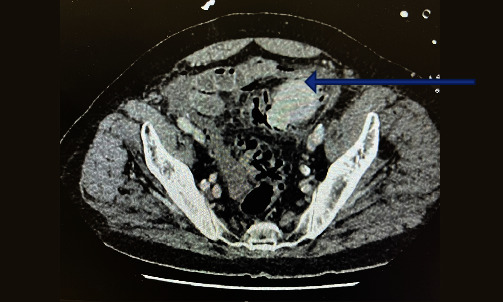
Large gallstone impacted in the sigmoid colon (see blue arrow).

On February 2023, he was diagnosed with sigmoid perforation secondary to gallstone impaction in the sigmoid colon (Figure 1). The patient underwent Hartmann's procedure due to sigmoid perforation and widespread feculent peritonitis. Given concerns about haemodynamic instability demonstrated during the operation, he transferred after the operation to the intensive care unit. The patient passed away on the third postoperative day due to complications of haemodynamic instability.

## 3. Discussion

This study demonstrates one of the rarest manifestations of gallstone ileus, namely, sigmoid perforation secondary to a large gallstone impaction in the sigmoid colon. The prevalence rate of female gender in patients with gallstone ileus is higher than that of males [2–4].

Both the management and outcome of sigmoid gallstone ileus depend on many factors, such as advanced old age, numerous related comorbidities, frailty status, and in the end, delayed presentation [12].

The most common presenting symptoms are constipation and abdominal pain. It has been reported that the duration of presenting symptoms is longer in the cohort of patients with sigmoid gallstone ileus compared with those with small bowel [11]. Probably, the late onset of distressing symptoms of nausea and vomiting is the cause of the late presentation [11]. In our case, the patient presented with one-week symptoms of severe constipation.

For the uncomplicated sigmoid gallstone, ileus is proposed following interventions. First, enterotolithotomy, means incision over the impacted gallstone and extraction. Second, milking of the impacted stone to the caecum and extraction through the hole of appendicectomy. In addition, it is reported successful laparoscopic management of colonic gallstone obstruction [11, 12]. Moreover, it has been reported successful treatment of large gallstones with endoscopic mechanical lithotripsy [13]. However, in the case of acutely unwell patients with colonic perforation, the treatment of choice is Hartmann's procedure. Given the fragility of such patient general anaesthesia and surgery may push the functional reserves to their limits [14]. Our patient passed away on the third postoperative day due to complications of haemodynamic instability.

Recent evidence demonstrates that laparoscopic cholecystectomy is safe in elderly patients. It has been reported that gallstone greater than 5 cm is more likely to cause colonic obstruction [15]. Therefore, patients with large gallstones should seriously be considered for elective cholecystectomy.

## 4. Conclusions

Patients diagnosed with uncomplicated sigmoid colonic obstruction can be managed with lithotripsy; if the procedure fails, milking of the stone in the caecum and extraction through the hole of appendicectomy avoids the complications of bowel resection. However, in the case of perforation and/or ischaemia, the surgical intervention must not be delayed.

## Data Availability

The authors declare that data supporting the findings of this study are available within the article.
